# Anti-CTLA-4 treatment suppresses hepatocellular carcinoma growth through Th1-mediated cell cycle arrest and apoptosis

**DOI:** 10.1371/journal.pone.0305984

**Published:** 2024-08-06

**Authors:** Hitomi Morihara, Tomomi Yamada, Yumi Tona, Marina Akasaka, Hirohisa Okuyama, Natsumi Chatani, Satomi Shinonome, Azumi Ueyama, Kenji Kuwabara, Yasushi Fujio

**Affiliations:** 1 Laboratory of Clinical Science and Biomedicine, Graduate School of Pharmaceutical Sciences, Osaka University, Suita, Osaka, Japan; 2 Laboratory for Bio-Drug Discovery, Shionogi & Co., Ltd., Osaka, Japan; Abu Dhabi University, UNITED ARAB EMIRATES

## Abstract

Inhibiting the cytotoxic T-lymphocyte-associated protein-4 (CTLA-4)-mediated immune checkpoint system using an anti-CTLA-4 antibody (Ab) can suppress the growth of various cancers, but the detailed mechanisms are unclear. In this study, we established a monoclonal hepatocellular carcinoma cell line (Hepa1-6 #12) and analyzed the mechanisms associated with anti-CTLA-4 Ab treatment. Depletion of CD4^+^ T cells, but not CD8^+^ T cells, prevented anti-CTLA-4 Ab-mediated anti-tumor effects, suggesting dependence on CD4^+^ T cells. Anti-CTLA-4 Ab treatment resulted in recruitment of interferon-gamma (IFN-g)-producing CD4^+^ T cells, called T-helper 1 (Th1), into tumors, and neutralization of IFN-g abrogated the anti-tumor effects. Moreover, tumor growth suppression did not require major histocompatibility complex (MHC)-I or MHC-II expression on cancer cells. *In vitro* studies showed that IFN-g can induce cell cycle arrest and apoptosis in tumor cells. Taken together, these data demonstrate that anti-CTLA-4 Ab can exert its anti-tumor effects through Th1-mediated cell cycle arrest and apoptosis.

## Introduction

Despite advancements in drug discovery, cancer remains one of the top 10 causes of death worldwide [1], suggesting that novel treatment methods are still needed. Immunotherapy has become a focus of cancer research. Immune checkpoint inhibitors, such as anti-cytotoxic T-lymphocyte-associated protein-4 (CTLA-4) antibodies (Abs), have been attracting attention as breakthrough anti-cancer therapies in recent years. Ipilimumab, a monoclonal Ab that inhibits CTLA-4 on T cells, became the world’s first approved immune checkpoint inhibitor in 2011 for treating advanced melanoma. Since then, it has been expanded to treat other various cancers, such as metastatic renal cell carcinoma, metastatic colorectal cancer, and hepatocellular carcinoma [2].

CTLA-4 is a T cell surface protein whose expression increases upon activation. It is known to bind to CD80/86 on antigen-presenting cells and inhibit T cell activation. Most of the extracellular suppressive function of CTLA-4 is mediated by regulatory T cells (Tregs) [3]. Therefore, inhibition of CTLA-4 has been thought to directly activate T cells and release T cell-mediated immunosuppression by Tregs, resulting in long-term anti-tumor effects. In fact, anti-CTLA-4 Abs have shown potent efficacy in preclinical studies. However, their clinical use has raised unexpected issues. For example, it is becoming clear that their efficacy is limited to a certain subgroup of cancer patients [4, 5]. In addition, their use in the clinic has been limited due to their strong side effects, such as severe diarrhea, colitis, and hypophysitis [4, 6]. Thus, a better understanding of the detailed mechanism underlying anti-CTLA-4 treatment efficacy may lead to the development of drugs with fewer side effects and potent anti-tumor effects.

Some studies have proposed that CD8^+^ T cells are involved in the anti-tumor effects of anti-CTLA-4 Abs and drug discovery targeting CD8^+^ T cells is progressing worldwide. On the other hand, the pathophysiological roles of CD4^+^ T cells in anti-CTLA-4 therapy remains to be fully elucidated. In the clinic, activation of CD4^+^ T cells by anti-CTLA-4 Abs has been observed [7]. In this study, a mouse tumor model Hepa1-6, which is sensitive to CD4^+^ T cell activity, was used to conduct mechanistic analysis of an anti-CTLA-4 Ab [8]. Our results revealed that IFN-g produced by T-helper 1 (Th1) plays an important role in the elimination of hepatocellular carcinoma mediated by an anti-CTLA-4 Ab. This highlights the importance of CD4^+^ T cells in tumor immunity.

## Materials and methods

### Mice

C57BL/6 mice and BALB/c mice were purchased from CLEA Japan, Inc. (Tokyo, Japan). They were kept under specific pathogen-free conditions. Cells were injected when the mice were 6–9 weeks old. All experiments were conducted in accordance with the Act on Welfare and Management of Animals in Japan and the Guide for the Care and Use of Laboratory Animals. The animal study protocol was approved by the Institutional Animal Care and Use Committee of Shionogi & Co., Ltd., which is accredited by AAALAC International. The mice were euthanatized by using carbon dioxide.

### Cell culture and chemicals

Murine tumor cell lines Hepa1-6, CT26. WT, and EMT6 were purchased from American Type Culture Collection (Manassas, VA, USA) and MB49 cells were purchased from EMD Millipore Corporation (Burlington, MA, USA). These cell lines were cultured in DMEM (Sigma-Aldrich, St. Louis, MO, USA) supplemented with 10% fetal bovine serum (FBS; Hyclone, Logan, UT, USA), 100 units/mL penicillin, and 100 μg/mL streptomycin (Nacalai tesque, Kyoto, Japan) at 37°C and 5% CO_2_. Mycoplasma-negative cells were purchased and used.

### Establishment of Hepa1-6 #12 cell lines

Hepa1-6 cells were harvested, and one to two million cells were injected subcutaneously into the mouse. Two months later, the tumor was retrieved and injected subcutaneously into another mouse. Following an additional two months, the tumor was collected and treated with collagenase. Single cell suspensions were seeded into a 96-well plate at a limiting dilution and several single colonies were selected. Clone #12 (Hepa1-6 #12) was selected for its high *in vitro* growth activity and stable *in vivo* viability.

### *In vivo* tumor experiments

Hepa1-6 #12 cells were harvested, and one to two million cells were suspended in PBS. Then these cells were resuspended in an equal volume of growth factor-reduced Matrigel (Corning, Corning, NY, USA). The prepared cell suspension was then injected subcutaneously into the right back of the mouse (day 0). Tumor volume was measured at least twice a week with a digital caliper. Mice were euthanized when tumor volume reached 2,000 mm^3^. In all studies, mice were injected with 200 μg anti-CTLA-4 Ab (cat. No. BE0164, clone 9D9; BioXcell, Lebanon, NH, USA) at intervals of 3 or 4 days from day 5. For the isotype control, 200 μg mouse IgG2b (cat. No. BE0086, clone MPC-11; BioXcell) was used. 250 μg anti-CD4 Ab (cat. No. BE0003-1, clone GK1.5; BioXcell) or 250 μg anti-CD8β Ab (cat. No. BE0223, clone 53–5.8; BioXcell) was used for T cell depletion, and 200 μg anti-CD193 Ab (cat. No. BE0316, clone 6S2-19-4; BioXcell) was used for eosinophil depletion. For IFN-g neutralization, 250 μg anti-IFN-g Ab (cat. No. I-438, clone H22; Leinco Technologies, St. Louis, MO, USA) or isotype (cat. No. I-140, clone PIP; Leinco Technologies) was used. All Abs were administered intraperitoneally as described in the figure legends.

### Antibodies

Abs for flow cytometry (FCM) or immunohistochemistry (IHC) were purchased from BioLegend (San Diego, CA, USA), eBioscience (Thermo Fisher Scientific, Waltham, MA, USA), BD Biosciences (Franklin Lakes, NJ, USA) and Cell Signaling Technology (Danvers, MA, USA). The Ab list is shown in Table 1.

**Table 1:** 

**Antibodies for FCM**			
**Protein**	**Clone**	**Manufacturer**	**Concentration**
Arg1	A1exF5	Invitrogen	1:100
CD3	145-2C11	BioLegend	1:200
CD4	RM4-5	BioLegend	1:100
CD8α	53–6.7	BioLegend	1:200
CD8α	53–6.7	BioLegend	1:200
CD11b	M1/70	eBioscience	1:200
CD19	6D5	BioLegend	1:200
CD45	30F-11	BioLegend	1:200
CD193	J073E5	BioLegend	1:200
CD326	G8.8	BioLegend	1:200
F4/80	BM8	BioLegend	1:200
Foxp3	FJK-16s	eBioscience	1:100
Gata3	TWAJ	eBioscience	1:100
H-2Db	KH95	BioLegend	1:200
H-2Kb	AF6-88.5	BioLegend	1:200
I-A/I-E	M5/114.15.2	BioLegend	1:200
IFNγ	XMG1.2	BioLegend	1:100
Ly6C	HK1.4	BioLegend	1:200
Ly6G	1A8	BioLegend	1:200
NK1.1	PK136	BioLegend	1:200
NOS2	CXNFT	eBioscience	1:100
Rorγt	B2D	eBioscience	1:100
Siglec-F	E50-2440	BD Biosciences	1:200
T-bet	4B10	BioLegend	1:100
TCR-β	H57-597	BioLegend	1:200
**Antibodies for IHC**			
**Protein**	**Clone**	**Manufacturer**	**Concentration**
CD4	D7D2Z	Cell Signaling Technology	1:400
CD8α	D4W2Z	Cell Signaling Technology	1:1600
CD31	MEC13.3	BioLegend	1:100
Foxp3	D6O8R	Cell Signaling Technology	1:1600
NG2	polyclonal	Merck Millipore	1:200
αSMA	D4K9N	Cell Signaling Technology	1:100

### Flow cytometry (FCM)

To analyze tumor infiltrating lymphocytes (TILs), tumor tissues were resected from mice, minced with scissors, then incubated in 1 mL of the tumor dissociation buffer (50 U/mL DNase I (Worthington Biochemical, Lakewood, NJ, USA) and 100 μg/mL Collagenase type IA (Sigma-Aldrich) in RPMI) for 40–60 minutes (min) at 37°C under continuous rotation. Cells were washed with FCM buffer (2% FBS, 5 mM EDTA, and 5 mM HEPES in Phenol red free Hank’s (Sigma-Aldrich)) and filtered through 100 μm nylon mesh. After being treated with red blood lysis buffer solution, cells were resuspended in 20% Percoll Plus (GE Healthcare, Chicago, IL, USA) to isolate tumor cells and TILs. After blocking with FcR blocking solution (Miltenyi Biotec, Bergisch Gladbach, Germany), each Ab was added at the concentration described in Supplementary Data1 and incubated for 30 min at 4°C in the dark. The Foxp3 staining kit (eBioscience) was used for intracellular staining. We used DAPI (Dojindo, Kumamoto, Japan), eBioscience^™^ Fixable Viability Dye eFluor^™^ 780 (Invitrogen) or the Zombie NIR Fixable Viability kit (BioLegend) to stain dead cells. To analyze cytokine production, cells were stimulated with 2 μL/mL Cell Stimulation Cocktail (Thermo Fisher Scientific) and BD Golgistop (BD Bioscience) for 4 hr at 37°C before FcR blocking. To detect expression of MHC-I in Hepa1-6 #12 cell lines *in vitro*, cells were stimulated with 100 ng/mL Recombinant Mouse IFN-g (carrier-free) (rMuIFN-g: BioLegend) for 48 hr at 37°C. Data were acquired using MACSQuant (Miltenyi Biotec), NovoCyte (Agilent Technologies, Santa Clara, CA, USA), or Spectral Cell Analyzer ID7000 (Sony, Tokyo, Japan).

### Immunohistochemistry (IHC)

To analyze the tumor microenvironment, tumor tissues were collected and fixed with 4% paraformaldehyde (Electron Microscopy Sciences, Hatfield, PA, USA) overnight at 4°C. Section preparation and staining for CD4, CD8, and FOXP3 were performed by Applied Medical Research Laboratory. For analyzing frozen sections, slides were washed with PBS to remove OCT compound and blocked with blocking buffer (5% goat serum and 0.3% TritonX-100 in PBS) for 1 hr, then incubated with primary Abs overnight at 4°C. After washing, the sections were incubated with secondary Abs for 1 hr at room temperature in the dark, then mounted with ProLong Diamond Antifade Mountant with DAPI (Thermo Fisher Scientific). All sections were analyzed with HALO Image Analysis software (Indica Labs, Albuquerque, NM, USA).

### Generation of β2m and MHC-II-KO Hepa1-6#12 cell lines using CRISPR/Cas9

To generate Hepa1-6 #12 cell lines lacking β2m or MHC-II expression, we used previously reported single-guide RNAs (sgRNAs) [9, 10]. The sgRNAs were subcloned into the GeneArt^®^ CRISPR Nuclease OFP Vector (Invitrogen). Cells were transfected with 2.5 μg plasmid DNA in a culture plate using Lipofectamine 3000 (Invitrogen) per the manufacturer’s protocol. Two days later, OFP vector-positive cells were single-cell sorted using a FACSAria-II Cell Sorter (Becton, Dickinson and Company, Franklin Lakes, NJ, USA) and further expanded. The sgRNA list is shown in Table 2.

**Table 2:** sgRNAs for CRISPR/Cas9.

Gene	Sequence
β2m	5’-TCGGCTTCCCATTCTCCGGT-3’
H2-Aa1	5’-GGAGGTGAAGACGACATTGA-3’

### RNA-sequencing

RNA was isolated using the RNeasy Mini Kit (Qiagen, Hilden, NRW, Germany). Isolated RNA was immediately converted to cDNA using PrimeSTAR MAX DNA Polymerase (Takara Bio, Shiga, Japan). Then, cDNA was cleaned up using ExoSap-IT Express (Thermo Fisher Scientific) and purified for sequencing using the BigDye Terminator v3.1 Cycle Sequencing Kit (Thermo Fisher Scientific). Sanger sequencing was then performed on the samples using a 3130xl genetic analyzer (Applied Biosystems, Thermo Fisher Scientific, Waltham, MA, USA) to confirm disruption of the target locus. The primer list is shown in Table 3.

**Table 3:** Primers for RNA-sequencing.

Gene	Direction	Sequence
β2m	Forward	5’-TGTGCAGAATGGGATGTGAC-3’
	Reverse	5’-GGCACCACAGATCAGTCTTTTGG-3
H2-Aa1	Forward	5’-CTGGCAACTTTGACGTCATC-3’
	Reverse	5’-TTTCTTCCTTCCCTCACTGG-3’

### Cell viability assay

Hepa1-6 #12 cells (1^10^3^ cells/well) were cultured with 100 ng/mL rMuIFN-g. 96 hr later, the percentages of viable cells were measured by WST-8 assay (Kishida Chemical, Osaka, Japan) per the manufacturer’s protocol. For apoptosis detection, Hepa1-6 #12 cells (1^10^4^ cells/well) were cultured with 100 ng/mL rMuIFN-g. After 96 hr, the cells were examined with Annexin V-FITC Apoptosis Detection Kit (eBioscience) per the manufacturer’s protocols.

### Cell cycle analysis

Hepa1-6 #12 cells (1^10^5^ cells/dish) were cultured with 100 ng/mL rMuIFN-g and incubated for 96 hr at 37°C. After PBS washes, the cells were fixed in cold 70% ethanol for 2–3 days at 4°C. After three PBS washes, the cells were then treated with 250 μg/mL Ribonuclease (Nippon gene, Tokyo, Japan) for 1 hr at 37°C. 50 μg/mL propidium iodide (PI; Sigma-Aldrich) was added and shielded from light for 30 min. The samples were measured with MACSQuant.

### Statistical analysis

Statistical analysis was performed using GraphPad Prism 8 software (GraphPad Software, Inc., San Diego, CA, USA). One-way ANOVA followed by Dunnett’s test, One-way ANOVA followed by Tukey’s test or Mann-Whitney test was used for *in vivo* comparisons and Welch’s t-test for *in vitro* comparisons. *P*-values < 0.05 were considered statistically significant.

## Results

### CD4^+^ T cells are essential for anti-CTLA-4 treatment against tumor growth in Hepa1-6 model

**Fig 1:**
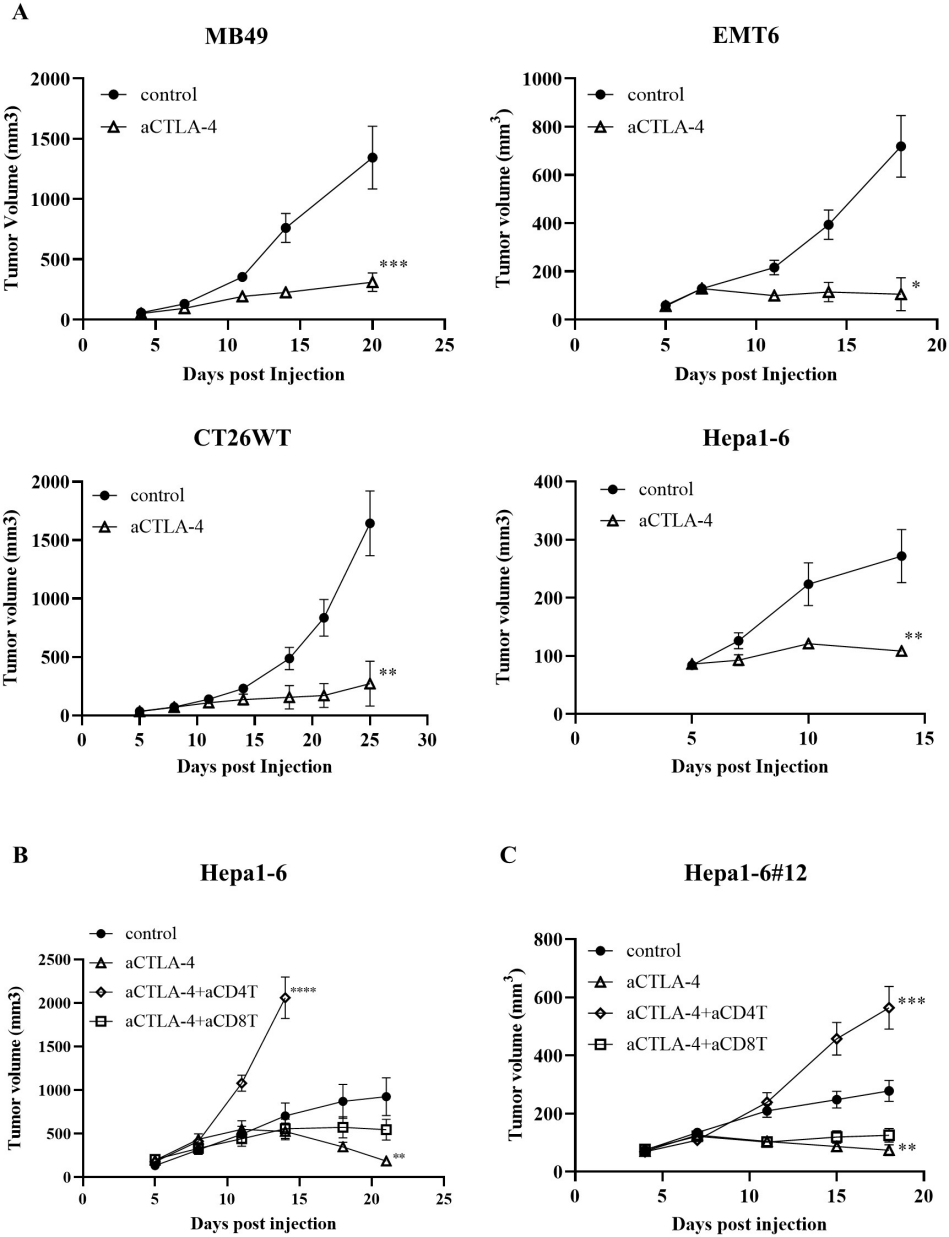
CD4^+^ T cells play a crucial role in immune checkpoint inhibitor treatment against tumor growth. Tumor growth of (A) MB49 (n = 7), EMT-6 (n = 5), CT26WT (n = 7) and Hepa1-6 (n = 5) tumors with or without anti-CTLA-4 Ab treatment (n = 7). The average tumor volume is shown as the mean ± standard error of the mean (SEM). * *P* < 0.05, ** *P* < 0.01, *** *P* < 0.001 by Mann-Whitney test at the endpoint. (B, C) Tumor growth of Hepa1-6 or Hepa1-6#12 tumors with or without anti-CTLA-4 Ab co-administered with a depleting Ab against CD4^+^ or CD8^+^ T cells. These depletion Abs were injected via intraperitoneal administration starting 2 days prior to tumor cell injection and continued weekly. The average tumor volume is shown as the mean ± SEM (n = 7–8). * *P* < 0.05, ** *P* < 0.01, *** *P* < 0.001 by one-way ANOVA followed by Dunnett’s test at the endpoint.

First, we tested the anti-tumor effects of the anti-CTLA-4 Ab in mouse models of bladder cancer (MB49), breast cancer (EMT6), colorectal cancer (CT26WT), and liver cancer (Hepa1-6). The results showed that the anti-CTLA-4 Ab had potent efficacy in all models (Fig 1A). To analyze the CD4^+^ T cell-mediated mechanism by anti-CTLA-4 Ab, this study focused on Hepa1-6 model, which is sensitive to CD4^+^ T cell activity [8]. We examined whether T cells are involved in the observed anti-tumor effects of anti-CTLA-4 Ab treatment. The depletion of CD4^+^ T cells prevented anti-tumor effects of the anti-CTLA-4 Ab, whereas depletion of CD8^+^ T cells had little effect in the Hepa1-6 model. This suggests that the efficacy of the anti-CTLA-4 Ab depends mainly on CD4^+^ T cells in this model (Fig 1B). We next generated a sub strain because the heterogeneity of Hepa1-6 cells was not suitable for further detailed mechanistic analysis using knocked out (KO) strains. Among the sub-strains generated, #12 was judged to be the most suitable for the analysis because of its *in vitro* growth ability and *in vivo* stability. Therefore, #12 was used in future studies. Similar to the parental strain, anti-CTLA-4 Ab efficacy was dependent on CD4^+^ T cell activity in this sub-strain (Fig 1C).

### The anti-CTLA-4 Ab can induce Th1 infiltration

**Fig 2:**
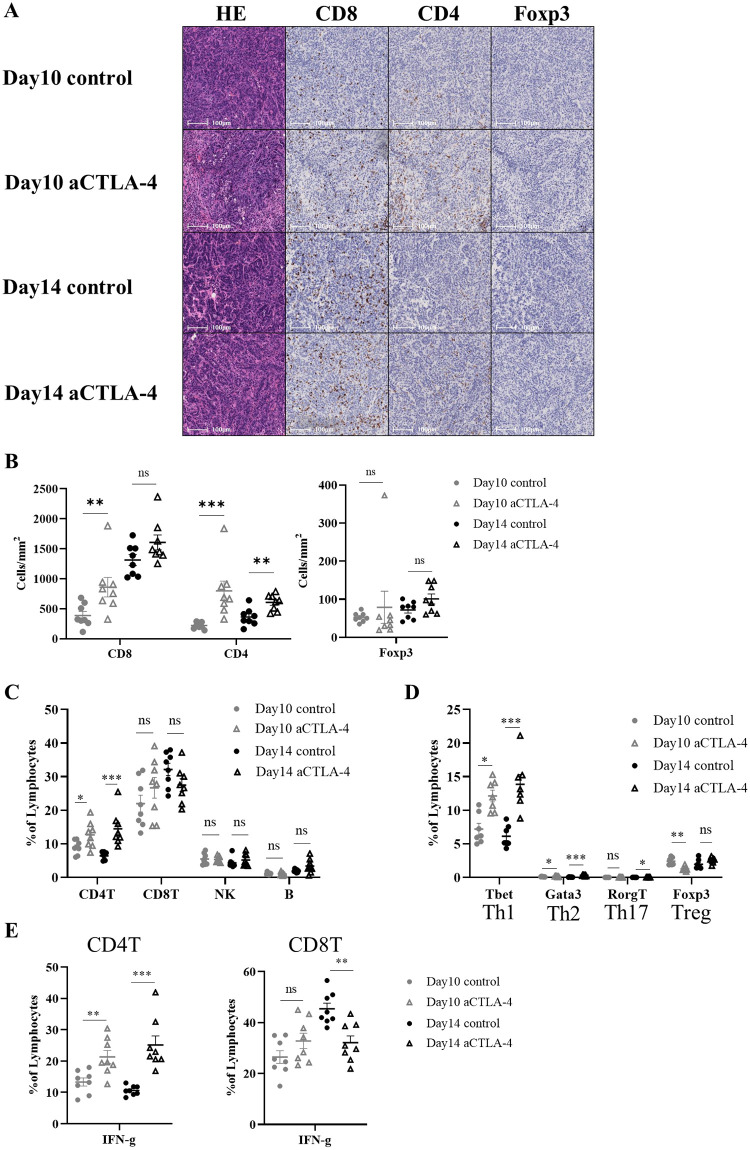
Anti-CTLA-4 Ab treatment induces Th1 cell infiltration. Hepa1-6#12 tumors were treated with a mouse IgG2b control Ab or anti-CTLA-4 Ab on days 5, 8, and 11. (A, B) Murine tumor sections were prepared on days 10 and 14 after injection. The sections were histologically and immunohistochemically analyzed by staining for CD8^+^, CD4^+^, and Foxp3^+^ cells. Images were analyzed using HALO software. (A) Representative images are shown. (B) Quantification of TILs. Each cell density is shown as the mean ± SEM (n = 8). ** *P* < 0.01, *** *P* < 0.001 by Mann-Whitney test. (C–E) TILs were analyzed on days 10 and 14 by FCM. Percentage of each cell is shown as the mean ± SEM (n = 7–8). * *P* < 0.05, ** *P* < 0.01, *** *P* < 0.001 by Mann-Whitney test.

Next, we analyzed tumor-infiltrating T cells in the Hepa1-6#12 model by immunohistochemistry (IHC) and Flow cytometry (FCM). Analysis of intratumoral TILs (iTILs) within the cancer cell nests using HALO software showed that administration of the anti-CTLA-4 Ab significantly increased the infiltration of CD4^+^ T cells into the tumor (Fig 2A and 2B). FCM analysis also revealed that administration of the anti-CTLA-4 Ab increased CD4^+^ T cell infiltration into the tumor (Fig 2C). However, no changes were observed in CD8^+^ T cell, natural killer (NK) cell, or B cell infiltration (Fig 2C, S1A Fig). Furthermore, Th1, Th2, Th17, and Treg markers (T-box expressed in T cells (T-bet), GATA binding protein 3 (GATA3), Retinoic acid receptor-related orphan receptor-gt (Rorgt), and Forkhead box P3 (Foxp3)) were stained for CD4^+^ T cell subset analysis. These results showed that many Th1, which release IFN-g, infiltrated into the tumor (Fig 2D and 2E, S1B Fig). Similar results were obtained for the parent Hepa1-6 model (S1C–S1E Fig).

### Hepa1-6 #12 tumor rejection induced by anti-CTLA-4 Ab treatment was independent of MHC-I and MHC-II expression on tumor cells

**Fig 3:**
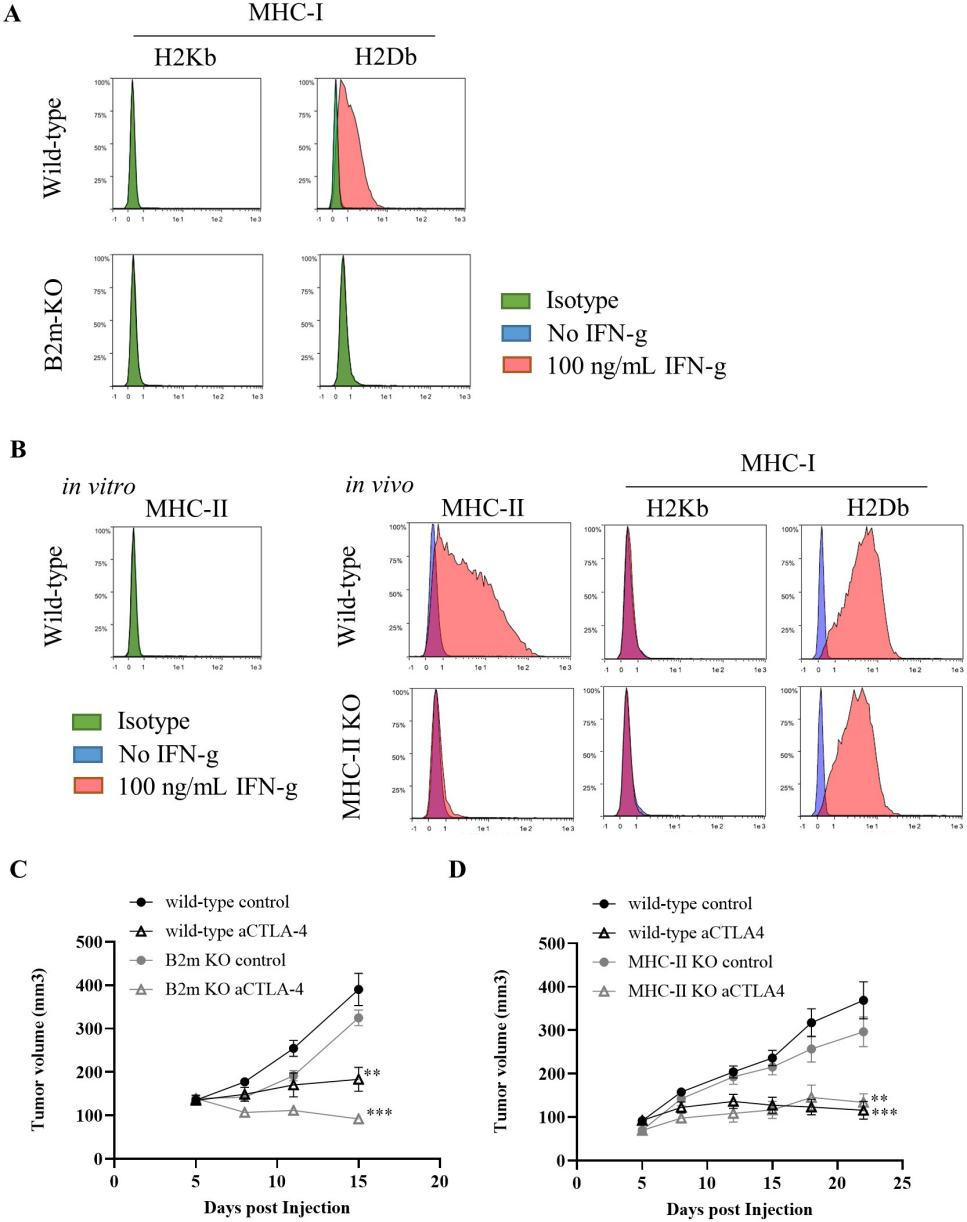
Hepa1-6 #12 tumor rejection induced by anti-CTLA-4 Ab treatment does not depend on MHC-I and MHC-II expression on tumor cells. (A) *In vitro* expression of MHC-I on wild-type or β2m KO Hepa1-6 #12 tumor cells. (B) *In vitro* expression of MHC-II on wild-type Hepa1-6 #12 tumor cells and *in vivo* expression of MHC-I and MHC-II evaluated 14 days after inoculation of wild-type or MHC-II KO Hepa1-6 #12 tumor cells. Tumor growth of (C) β2m- or (D) MHC-II-KO Hepa1-6 #12 tumors with or without anti-CTLA-4 Ab treatment on days 5, 8, 11, and 15. The average tumor volume is shown as the mean ± SEM (n = 7–8). ** *P* < 0.01, *** *P* < 0.001 by Mann-Whitney test at the endpoint. Data are representative of studies with two different KO clones.

One possible anti-tumor mechanism mediated by CD4^+^ T cells is CD4^+^ T cells recognize MHC-II on cancer cells and eliminate the tumor [11]. To determine whether the antitumor effects of the anti-CTLA-4 Ab depends on direct MHC-mediated recognition of cancer cells by T cells, we generated Hepa1-6 #12 cells with beta-2 microglobulin (β2m) or MHC-II expression KO using the CRISPR-Cas9 technique. The deletion of MHC-I in B2M KO cells was confirmed as follows (Fig 3A): In wild type Hepa1-6 #12 cells, both H2Kb and H2Db, MHC-I markers, are expressed only at undetectable levels *in vitro*; however, H2Db, but not H2Kb, was increased by the stimulation with IFN-g. In contrast, neither H2Kb nor H2Db was not detected in B2M KO cells either in the presence or absence of IFN-g *in vitro*. Since MHC-II was not detected in wild type Hepa1-6 #12 cells either in the presence or absence of IFN-g *in vitro*, we examined whether MHC-II expression was upregulated *in vivo* (Fig 3B). The expression of MHC-II was upregulated in wild type Hepa1-6 #12 cells *in vivo*, while MHC-II KO cells failed to express MHC-II, indicating that MHC-II was successfully ablated in MHC-II KO cells. The KO strains exhibited growth activity comparable to that of the wild-type #12 (Fig 3C and 3D). Anti-CTLA-4 Ab exerted its anti-tumor effects in mice transplanted with either KO cell line. We also obtained comparable results with another KO cell line (data not shown). These results indicate that the expression of MHC-I and MHC-II on cancer cells is not necessary for anti-CTLA-4 Ab treatment.

### IFN-g contributes to the rejection of Hepa1-6 #12 tumor cells by the anti-CTLA-4 Ab

**Fig 4:**
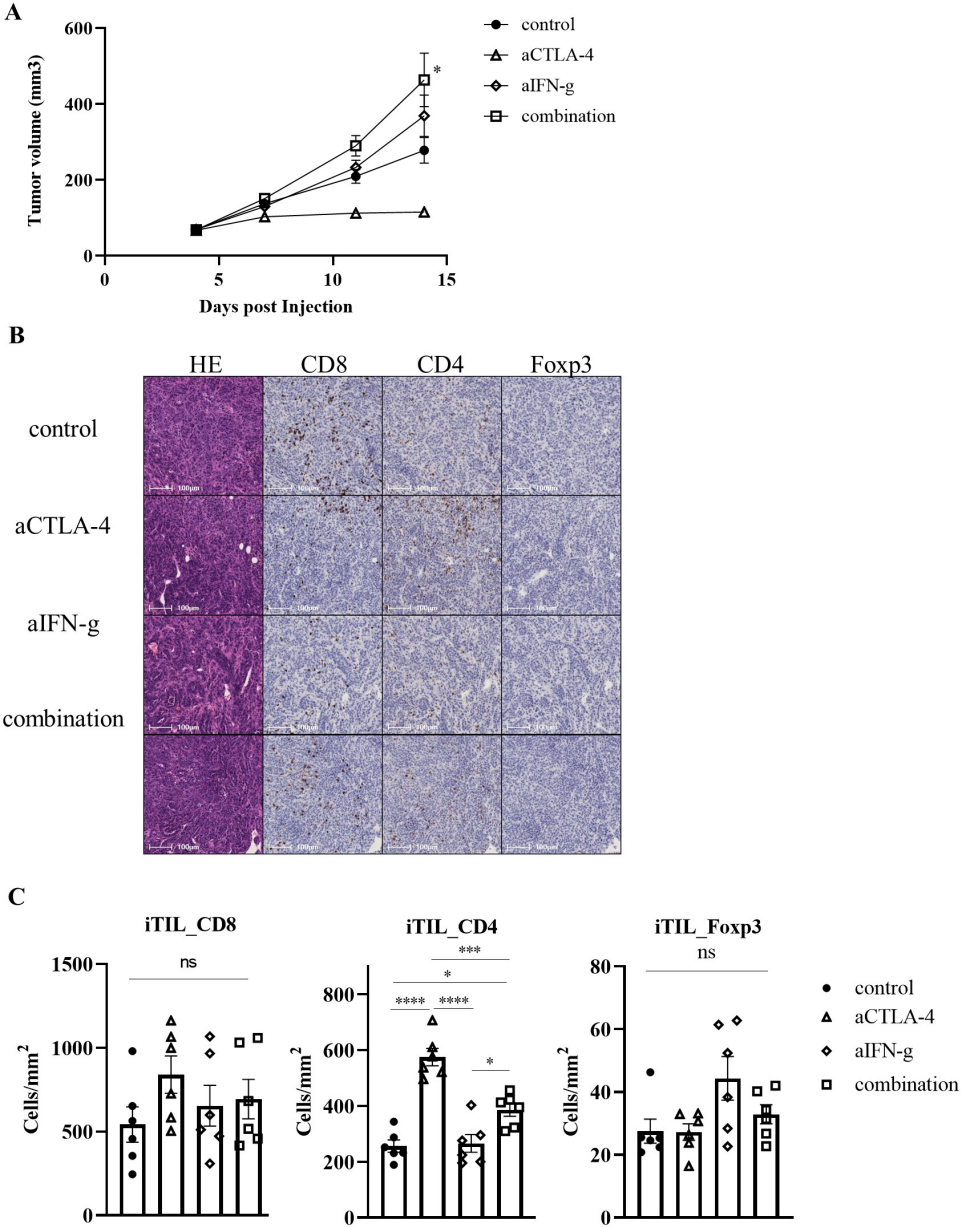
IFN-g is important for rejection of Hepa1-6 #12 tumors by anti-CTLA-4 Ab. (A) Tumor growth of Hepa1-6 #12 tumors with control or anti-CTLA-4 Ab treatment on days 5, 8, and 11 co-administered with IFN-g neutralizing Ab on days 3, 7, and 10. The average tumor volume is shown as the mean ± SEM (n = 8). * *P* < 0.05, ** *P* < 0.01 by one-way ANOVA followed by Dunnett’s test at the endpoint. (B, C) Murine tumor sections were prepared on day 11. The sections were histologically and immunohistochemically analyzed by staining for CD8^+^, CD4^+^, and Foxp3^+^ cells. Images were analyzed using HALO software. (B) Representative images are shown. (C) Quantification of TILs. Each cell density is shown as the mean ± SEM (n = 6). * *P* < 0.05, ** *P* < 0.01, *** *P* < 0.001, **** *P* < 0.0001 by one-way ANOVA followed by Tukey’s test.

Next, we examined the role of IFN-g produced by Th1 on tumor elimination by anti-CTLA-4 Ab. IFN-g neutralizing Ab could negate the efficacy of anti-CTLA-4 Ab treatment (Fig 4A, S2A Fig). In addition, the numbers of tumor infiltrated CD4^+^, CD8^+^, and Foxp3^+^ cells were evaluated by IHC. CD4^+^ cells, which had infiltrated into the tumor following anti-CTLA-4 Ab administration, showed a significant decrease in infiltration ability when combined with the IFN-g neutralizing Ab. However, treatment with the IFN-g neutralizing Ab alone showed no difference in CD4^+^ cell infiltration when compared with the controls (Fig 4B and 4C). Furthermore, treatment with the anti-CTLA-4 or IFN-g neutralizing Ab had no effects on CD8^+^ or Foxp3^+^ cell infiltration into the tumor. These results indicate that IFN-g produced by CD4^+^ T cells can accelerate infiltration of CD4^+^ cells during the tumor cell elimination process by the anti-CTLA-4 Ab.

### IFN-g can induce cell cycle arrest and apoptosis in Hepa1-6 #12 tumor cells

**Fig 5:**
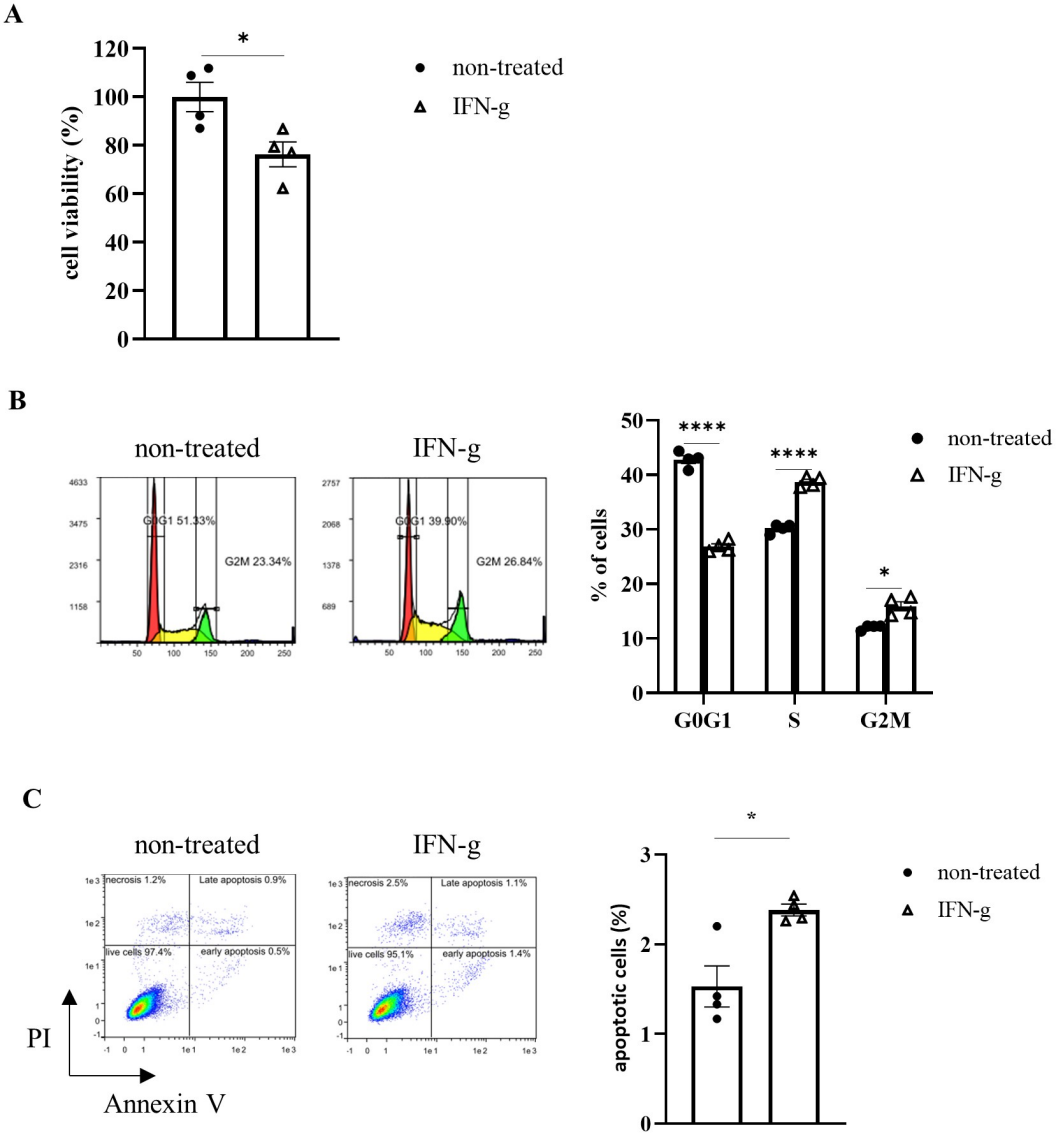
IFN-g can induce cell cycle arrest and apoptosis in Hepa1-6 #12 cells. (A) Analysis of cell viability was performed by WST-8 assays after IFN-g addition. (B) Cell cycle progression was evaluated by FCM after IFN-g addition. (C) Apoptotic cells were evaluated by FCM using Apoptosis detection kit after IFN-g addition. Data are shown as the mean ± SEM (n = 4). * *P* < 0.05, ****P < 0.0001 by Welch’s t-test.

Biological activities of IFN-g produced by CD4^+^ T cells have been reported to include the following: 1) enhanced anti-tumor activity through activation/anti-tiredness of CD8^+^ T cells [12], 2) enhanced inflammatory response through immune cells such as macrophages [13], 3) enhanced antigen presentation to cancer cells and growth inhibition [14], and 4) anti-tumor effects through regulation of the tumor blood vessels [15]. Next, we examined the contribution of myeloid cells, such as macrophages. Intratumor infiltration of macrophages, monocytic myeloid-derived suppressor cells (Mo-MDSCs), granulocytic MDSCs (Gr-MDSCs), and eosinophils were evaluated after administration of anti-CTLA-4 Ab (S3A and S3B Fig). Eosinophils infiltrated into the tumor after administration of the anti-CTLA-4 Ab, but depletion of eosinophils by anti-CD193 Ab treatment did not cancel the anti-tumor effect of the anti-CTLA-4 Ab (S3C Fig). M1 or M2 macrophage infiltration was also examined, but no changes that might contribute to the drug effect of the anti-CTLA-4 Ab were observed. This suggests that eosinophils and macrophages may not be involved in the drug efficacy. The effects of Ab treatment on blood vessels were also examined, but again no changes were observed (S4A–S4C Fig). Therefore, we addressed the possibility that IFN-g can directly act on cancer cell cycles. Since it was difficult to evaluate cancer cell apoptosis *in vivo* due to technical difficulty, we examined the effect of anti-CTLA-4 on Hepa1-6 cells *in vitro*. Recombinant IFN-g (100 ng/mL) was added to cultured Hepa1-6 #12 cells, then cell viability assays were performed 96 hours (hr) later (Fig 5A). The percentage of viable cells decreased by IFN-g treatment. Furthermore, IFN-g could induce cell cycle arrest in cancer cells, as well as apoptosis (Fig 5B and 5C). These results suggest that Th1 activated by anti-CTLA-4 Ab treatment exert anti-tumor effects by producing IFN-g and this can affect the cell cycle of cancer cells. Similar result was obtained for the parent Hepa1-6 (S5A Fig).

## Discussion

To investigate the mechanism of action of anti-CTLA-4 Ab, with a particular focus on CD4^+^ T cells, we performed a detailed analysis using the CD4^+^ T cell-sensitive Hepa1-6 model in this study. We showed that the anti-tumor effects of anti-CTLA-4 therapy were dependent on CD4^+^ T cells, but not CD8^+^ T cells in this model. Our data also suggested that anti-CTLA-4 Ab treatment led to Th1 accumulation in tumors, and IFN-g produced by these cells could induce cell cycle arrest and apoptosis in tumor cells, thereby possibly leading to tumor elimination. Moreover, this mechanism of action did not require MHC-I or MHC-II expression on cancer cells, suggesting that Th1-mediated anti-tumor effect of anti-CTLA-4 Ab may not be due to direct killing by CD4^+^ T cells. Importantly, no decrease in Treg abundance was observed in our experiments, although Treg depletion by anti-CTLA-4 Ab treatment has been reported to contribute to tumor elimination [16, 17]. Therefore, it could be proposed that this anti-CTLA-4 Ab can exhibit anti-tumor effects, at least partially, by enhancing Th1 function without depletion of Tregs. The clinically used anti-CTLA-4 Abs, ipilimumab and tremelimumab, have different Ab isotypes, with ipilimumab being a fully human IgG1 Ab and tremelimumab being a fully human IgG2 Ab. Although IgG1 Abs are known to mediate Ab-dependent cellular cytotoxicity (ADCC) more effectively than IgG2 Abs based on their binding affinities for human Fc receptors, ipilimumab and tremelimumab have similar response rates. Thus, Wei et al. suggested that the efficacy of anti-CTLA-4 Abs is derived from an enhancement of effector function rather than depletion [3], which is consistent with our findings. It has been reported that drug administration induces changes in Treg function, i.e. release of immunosuppressive mechanisms, increased CD8^+^ T intratumoral infiltration and activation of CD8^+^ T in the same liver cancer model (Hepa1-6) [18]. Since little activation of CD8^+^ T or increase in intra-tumor infiltration by anti-CTLA-4 Ab was observed in our model, it is unlikely that Treg function was altered by anti-CTLA-4 Ab.

In recent years, studies have reported that anti-CTLA-4 Ab administration can induce eosinophil accumulation and normalize blood vessels in breast cancer model mice [19]. In the present study, we observed an accumulation of eosinophils in tumors following administration of the anti-CTLA-4 Ab, but these cells were not involved in drug efficacy. The eosinophil accumulation observed here was possibly associated with a slight increase in Th2 cell abundance induced by anti-CTLA-4 Ab treatment. In addition, many studies have reported that IFN-g is involved in the pathophysiological regulation of tumor blood vessels, leading to various discussions that tumors can be eliminated by inhibiting angiogenesis [20, 21], regressing the vasculature [22], or normalizing blood vessels [15]. In this study, we also evaluated blood vessel density and normalization, but no changes were observed. At this time, we cannot completely explain why our findings are not fully consistent with the previous reports described above. However, the anti-CTLA-4 Ab possibly exhibits anti-tumor effects through multiple mechanisms that depend on the specific experimental conditions. Although Hepa1-6 model was used in this study, it has also been shown that CD4^+^ T cells, but not CD8^+^ T cells, are important for the efficacy of ICIs in MCB6C [9]. While the mouse model cannot be directly applied to human liver or bladder cancer, the results demonstrated that there is at least an immune environment in which CD4^+^ T cells are a key member of the tumor elimination mechanism. Although clinical data on CD4^+^ T cells in immunotherapy are limited, the therapeutic potential of CD4^+^ T cells has been demonstrated in cholangiocarcinoma and melanoma [23]. We believe that the mechanism discovered in this study works in these cancers and as a result, therapeutic effects may be seen.

Anti-CTLA-4 Ab therapy can cause side effects through inducing autoimmune complications by blocking the B7-CTLA-4 interaction [6]. The data presented here demonstrated that Th1 play an important role in the pharmacological mechanism of anti-CTLA-4 Ab-mediated actions in a liver cancer model. Therefore, differentiating naïve CD4^+^ T cells into Th1 without blocking the B7-CTLA-4 interaction may potentially lead to a reduction in the clinically observed side effects while still maintaining strong anti-tumor efficacy. Naïve CD4^+^ T cell differentiation into Th1 is reportedly mediated by certain cytokines, such as interleukin (IL)-12 and IFN-g [24], as well as by suppressed expression of *Gfi1* and *SOCS1* [25–27] and these may be safer, more effective targets. Notably, treatment with an IFN-g neutralizing Ab suppressed CD4^+^ cell, presumably CD4^+^ T cells, infiltration into tumors treated with the anti-CTLA-4 Ab, providing evidence that IFN-g produced from tumor-infiltrating Th1 can accelerate the infiltration of CD4^+^ T cells.

In conclusion, by using this liver tumor cell transplantation model, we demonstrated that anti-CTLA-4 Ab exhibits anti-tumor effects via the Th1/IFN-g axis. IFN-g may not only affect the cell cycle of cancer cells, but also enhance further infiltration of CD4^+^ T cells. Modulation of the Th1/IFN-g pathway may therefore be a novel therapeutic strategy for fighting cancer.

## Supporting information

S1 Fig Parent Hepa1-6 cells has a similar profile to the sub strain #12. (A) Gating strategies to detect lymphocytes. (B) Gating strategies to detect CD4^+^ T cell subsets. (C–E) Hepa1-6 tumors were treated with a mouse IgG2b control Ab or anti-CTLA-4 Ab on days 5, 8, and 11. (C) Murine tumor sections were prepared on days 10 and 14 after injection. The sections were histologically and immunohistochemically analyzed by staining for CD8^+^, CD4^+^, and Foxp3^+^ cells. Images were analyzed using HALO software. Quantification of TILs. Each cell density is shown as the mean ± SEM (n = 5). * P < 0.05, ** P < 0.01 by Mann-Whitney test. (D, E) TILs were analyzed on days 10 and 14 by FCM. Percentage of each cell is shown as the mean ± SEM (n = 5). * P < 0.05, ** P < 0.01 by Mann-Whitney test.
(TIF)

S2 Fig The anti-CTLA-4 Ab has no effect when IFN-g is neutralized. (A) Tumor growth of Hepa1-6 #12 tumors with or without anti-CTLA-4 Ab on days 5, 8, and 11 co-administered with an IFN-g depleting Ab on days 3, 7, and 10. Quantification of tumor volume at day 11. Data are shown as the mean ±SEM (n = 8). * *P* < 0.05 by one-way ANOVA followed by Dunnett’s test.
(TIF)

S3 Fig Myeloid cells have a low contribution to the efficacy of anti-CTLA-4 Abs. Tumors were treated with anti-CTLA-4 Ab or isotype control on days 5, 7, and 10. (A) Gating strategies to detect myeloid cells. (B) Tumor infiltrated myeloid cells were analyzed on days 10 and 14 by FCM. NOS2-producing macrophages are referred to as "M1" and arginase 1-producing macrophages are referred to as "M2". Percentage of each cell is shown as the mean ± SEM (n = 7). * *P* < 0.05, *** *P* < 0.001 by Mann-Whitney test. (C) Tumor growth of Hepa1-6 #12 tumors with or without anti-CTLA-4 Ab treatment on days 5, 8, 11, 14, and 18 co-administered with an eosinophil depleting Ab on days 4, 11, and 18. The average tumor volume is shown as the mean ± SEM (n = 7). **** *P* < 0.0001 by one-way ANOVA followed by Dunnett’s test at the endpoint.
(TIF)

S4 Fig The anti-CTLA-4 Ab does not affect blood vessel maturation. Tumors were treated with or without anti-CTLA-4 Ab treatment on days 5 and 8 co-administered with an IFN-g depleting Ab on days 4 and 7. Murine tumor sections were prepared on day 11 and analyzed using immunofluorescence staining for CD31, alpha-smooth muscle actin (α-SMA), and neuron-glial antigen 2 (NG2). Images were analyzed using HALO software. (A, B) Representative images are shown. (C) Quantification of mature blood vessel. Data are shown as the mean ± SEM (n = 6). * *P* < 0.05 by one-way ANOVA followed by Tukey’s test.
(TIF)

S5 Fig IFN-g decrease cell viability of Hepa1-6 cells. (A) Analysis of cell viability was performed by WST-8 assays after IFN-g addition. Data are shown as the mean ± SEM (n = 4). * *P* < 0.05 by Welch’s t-test.
(TIF)

S1 Raw data (XLSX)

## References

[1] World Health Organization (WHO). Global Health Estimates 2020: Deaths by Cause, Age, Sex, by Country and by Region, 2000–2019. WHO; 2020. Accessed December 11, 2020. https://www.who.int/data/gho/data/themes/mortality-and-global-health-estimates.

[2] Bagchi Sreya, Yuan Robert and Engleman Edgar G: Immune Checkpoint Inhibitors for the Treatment of Cancer: Clinical Impact and Mechanisms of Response and Resistance. Annu Rev Pathol: 16:223–249, 2021. doi: 10.1146/annurev-pathol-042020-042741 33197221

[3] Wei Spencer C, Duffy Colm R and Allison James P: Fundamental Mechanisms of Immune Checkpoint Blockade Therapy. Cancer Discov: (9): 1069–86, 2018. doi: 10.1158/2159-8290.CD-18-0367 30115704

[4] Seidel Judith A, Otsuka Atsushi and Kabashima Kenji: Anti-PD-1 and Anti-CTLA-4 Therapies in Cancer: Mechanisms of Action, Efficacy, and Limitations. Front Oncol: 8:86, 2018. doi: 10.3389/fonc.2018.00086 29644214 PMC5883082

[5] Siu Lillian L, Ivy S Percy, Dixon Erica L, Gravell Amy E, Reeves Steven A and Rosner Gary L: Challenges and Opportunities in Adapting Clinical Trial Design for Immunotherapies. Clin Cancer Res: 23(17); 4950–8, 2017. doi: 10.1158/1078-0432.CCR-16-3079 28864723 PMC5669041

[6] Karimi Amirali, Alilou Sanam and Mirzaei Hamid Reza: Adverse Events Following Administration of Anti-CTLA4 Antibody Ipilimumab. Front Oncol: 11:624780, 2021. doi: 10.3389/fonc.2021.624780 33767992 PMC7985548

[7] Chrysoula I Liakou Ashish Kamat, Derek Ng Tang Hong Chen, Sun Jingjing, Troncoso Patricia, et al: CTLA-4 blockade increases IFNgamma-producing CD4+ICOShi cells to shift the ratio of effector to regulatory T cells in cancer patients. Proc Natl Acad Sci U S A: 105(39):14987–92, 2008. doi: 10.1073/pnas.0806075105 18818309 PMC2567480

[8] Jin Ying, An Xiaoyu, Mao Binchen, Sun Ruilin, Kumari Rajendra, Chen Xiaobo, et al: Different syngeneic tumors show distinctive intrinsic tumor-immunity and mechanisms of actions (MOA) of anti-PD-1 treatment. Sci Rep: 12(1):3278, 2022. doi: 10.1038/s41598-022-07153-z 35228603 PMC8885837

[9] Sato Yuji, Bolzenius Jennifer K., Eteleeb Abdallah M., Su Xinming, et al: CD4^+^ T cells induce rejection of urothelial tumors after immune checkpoint blockade. JCI Insight: 3(23):e121062, 2018. 30518683 10.1172/jci.insight.121062 PMC6328023

[10] Freeman Andrew J., Vervoort Stephin J., Ramsbottom Kelly M., Kelly Madison J., Michie Jessica, Pijpers Lizzy, et al: Natural Killer Cells Suppress T Cell-Associated Tumor Immune Evasion. Cell Rep: 28(11):2784–2794, 2019. doi: 10.1016/j.celrep.2019.08.017 31509742

[11] Haabeth Ole Audun Werner, Tveita Anders Aune, Fauskanger Marte, Schjesvold Fredrik, Lorvik Kristina Berg, Hofgaard Peter O, et al: How Do CD4(+) T Cells Detect and Eliminate Tumor Cells That Either Lack or Express MHC Class II Molecules? Front Immunol: 5:174, 2014. doi: 10.3389/fimmu.2014.00174 24782871 PMC3995058

[12] Aubert Rachael D., Kamphorst Alice O., Sarkar Surojit, Vezys Vaiva, Ha Sang-Jun, Barber Daniel L., et al: Antigen-specific CD4 T-cell help rescues exhausted CD8 T cells during chronic viral infection. Proc Natl Acad Sci U S A: 108(52):21182–7, 2011. doi: 10.1073/pnas.1118450109 22160724 PMC3248546

[13] Biswas Subhra K and Mantovani Alberto: Macrophage plasticity and interaction with lymphocyte subsets: cancer as a paradigm. Nat Immunol: (10):889–96, 2010. doi: 10.1038/ni.1937 20856220

[14] Braumüller Heidi, Wieder Thomas, Brenner Ellen, Aßmann Sonja, Hahn Matthias, Alkhaled Mohammed, et al: T-helper-1-cell cytokines drive cancer into senescence. Nature: 494(7437):361–5, 2013. doi: 10.1038/nature11824 23376950

[15] Tian Lin, Goldstein Amit, Wang Hai, Hin Ching Lo Ik Sun Kim, Welte Thomas, et al: Mutual Regulation of Tumor Vessel Normalization an Immunostimulatory Reprogramming. Nature: 544(7649):250–254, 2017. 28371798 10.1038/nature21724 PMC5788037

[16] Du Xuexiang, Tang Fei, Liu Mingyue, Su Juanjuan, Zhang Yan, Wu Wei, et al: A reappraisal of CTLA-4 checkpoint blockade in cancer immunotherapy. Cell Res: (4): 416–432, 2018. doi: 10.1038/s41422-018-0011-0 29472691 PMC5939050

[17] Willsmore Zena N., Coumbe Ben G. T., Crescioli Silvia, Reci Sara, Gupta Ayushi, Harris Robert J., et al: Combined anti-PD-1 and anti-CTLA-4 checkpoint blockade: Treatment of melanoma and immune mechanisms of action. Eur J Immunol: (3):544–556, 2021. doi: 10.1002/eji.202048747 33450785

[18] Ueyama Azumi, Nogami Wataru, Nashiki Kunitaka, Haruna Miya, Miwa Hiroto, Hagiwara Masaki, et al: Immunotherapy Targeting CCR8+ Regulatory T Cells Induces Antitumor Effects via Dramatic Changes to the Intratumor CD8+ T Cell Profile. J Immunol: 211(4): 673–682, 2023. doi: 10.4049/jimmunol.2300067 37350632

[19] Zheng Xichen, Zhang Naidong, Qian Long, Wang Xuexiang, Fan Peng, Kuai Jiajie, et al: CTLA4 blockade promotes vessel normalization in breast tumors via the accumulation of eosinophils. Int J Cancer: 146(6):1730–1740, 2020. doi: 10.1002/ijc.32829 31840816

[20] Qin Z and Blankenstein T: CD4^+^ T Cell–Mediated Tumor Rejection Involves Inhibition of Angiogenesis that Is Dependent on IFNγ Receptor Expression by Nonhematopoietic Cells. Immunity: (6):677–86, 2000. 10.1016/s1074-7613(00)80218-6 10894167

[21] Beatty G and Paterson Y: IFN-γ-Dependent Inhibition of Tumor Angiogenesis by Tumor-Infiltrating CD4^+^ T Cells Requires Tumor Responsiveness to IFN-γ. J Immunol: 166(4):2276–82, 2001. 11160282 10.4049/jimmunol.166.4.2276

[22] Kammertoens Thomas, Friese Christian, Arina Ainhoa, Idel Christian, Briesemeister Dana, Rothe Michael, et al: Tumour ischaemia by interferon-γ resembles physiological blood vessel regression. Nature: 545(7652):98–102, 2017. 28445461 10.1038/nature22311 PMC5567674

[23] Poncette Lucia, Bluhm Julia and Blankenstein Thomas: The role of CD4 T cells in rejection of solid tumors. Curr Opin Immunol: 74: 18–24, 2022. doi: 10.1016/j.coi.2021.09.005 34619457 PMC8933281

[24] Swain Susan L, McKinstry K Kai and Strutt Tara M: Expanding roles for CD4+ T cells in immunity to viruses. Nat Rev Immunol: 12(2):136–48, 2012. 22266691 10.1038/nri3152 PMC3764486

[25] Suzuki Junpei, Maruyama Saho, Tamauchi Hidekazu, Kuwahara Makoto, Horiuchi Mika, Mizuki Masumi, et al: Gfi1, a transcriptional repressor, inhibits the induction of the T helper type 1 programme in activated CD4 T cells. Immunology: 147(4):476–87, 2016. doi: 10.1111/imm.12580 26749286 PMC4799889

[26] Tanaka Kentaro, Ichiyama Kenji, Hashimoto Masayuki, Yoshida Hideyuki, Takimoto Tomohito, Takaesu Giichi, et al: Loss of Suppressor of Cytokine Signaling 1 in Helper T Cells Leads to Defective Th17 Differentiation by Enhancing Antagonistic Effects of IFN-γ on STAT3 and Smads. J Immunol: 180(6):3746–56, 2008. 18322180 10.4049/jimmunol.180.6.3746

[27] Yoshimura Akihiko, Suzuki Mayu, Sakaguchi Ryota, Hanada Toshikatsu and Yasukawa Hideo: SOCS, inflammation, and autoimmunity. Front Immunol: 3:20, 2012. doi: 10.3389/fimmu.2012.00020 22566904 PMC3342034

