# Supplementary figures and images for: Anti-CTLA-4 treatment suppresses hepatocellular carcinoma growth through Th1-mediated cell cycle arrest and apoptosis
Source: PLoS One. 2024 Aug 6;19(8):e0305984. doi: 10.1371/journal.pone.0305984 (PMC11302986; doi:10.1371/journal.pone.0305984)

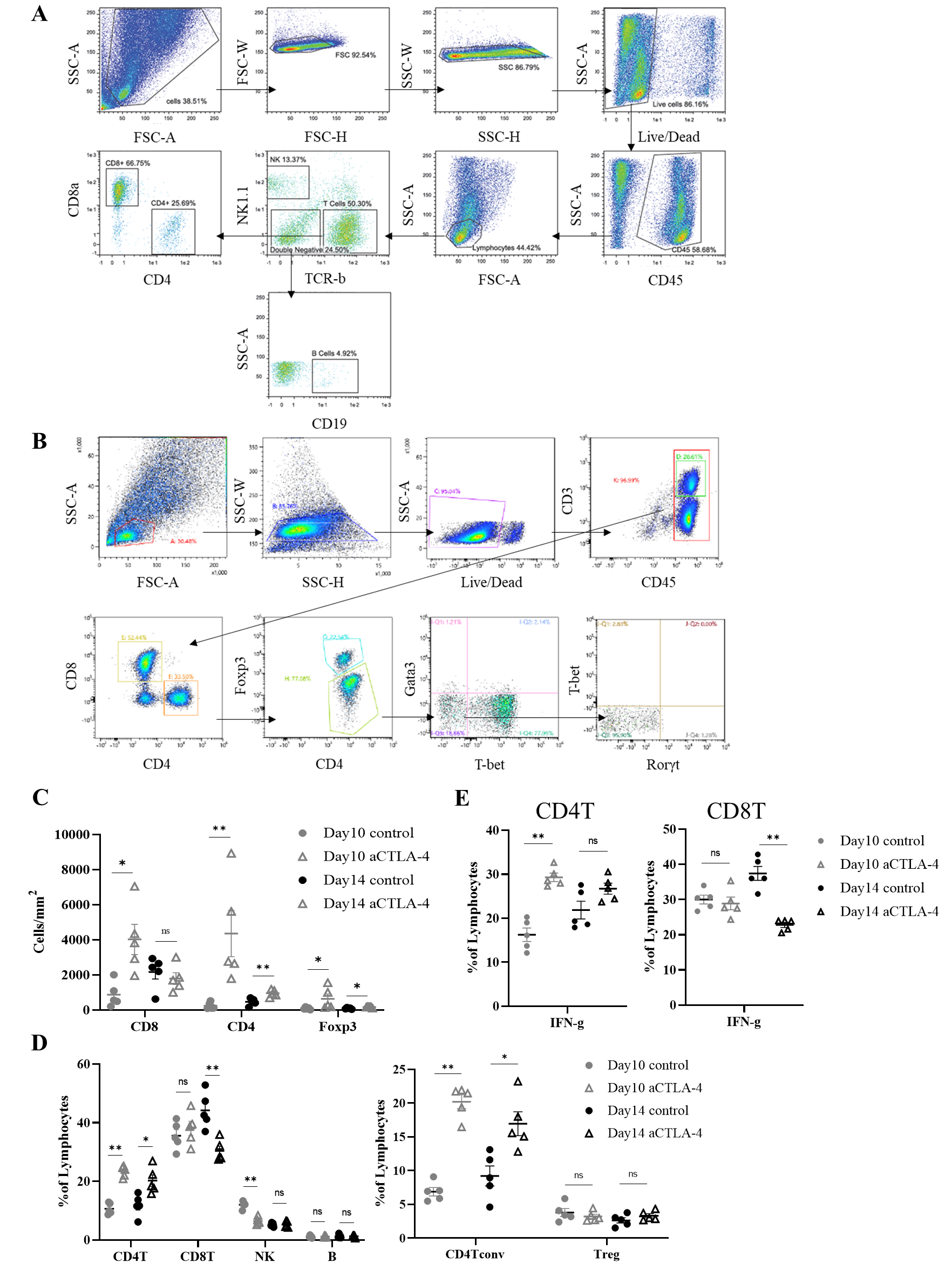

Supplement: S1 Fig — (A) Gating strategies to detect lymphocytes. (B) Gating strategies to detect CD4+ T cell subsets. (C–E) Hepa1-6 tumors were treated with a mouse IgG2b control Ab or anti-CTLA-4 Ab on days 5, 8, and 11. (C) Murine tumor sections were prepared on days 10 and 14 after injection. The sections were histologically and immunohistochemically analyzed by staining for CD8+, CD4+, and Foxp3+ cells. Images were analyzed using HALO software. Quantification of TILs. Each cell density is shown as the mean ± SEM (n = 5). * P < 0.05, ** P < 0.01 by Mann-Whitney test. (D, E) TILs were analyzed on days 10 and 14 by FCM. Percentage of each cell is shown as the mean ± SEM (n = 5). * P < 0.05, ** P < 0.01 by Mann-Whitney test. (TIF) [file pone.0305984.s001.tif]

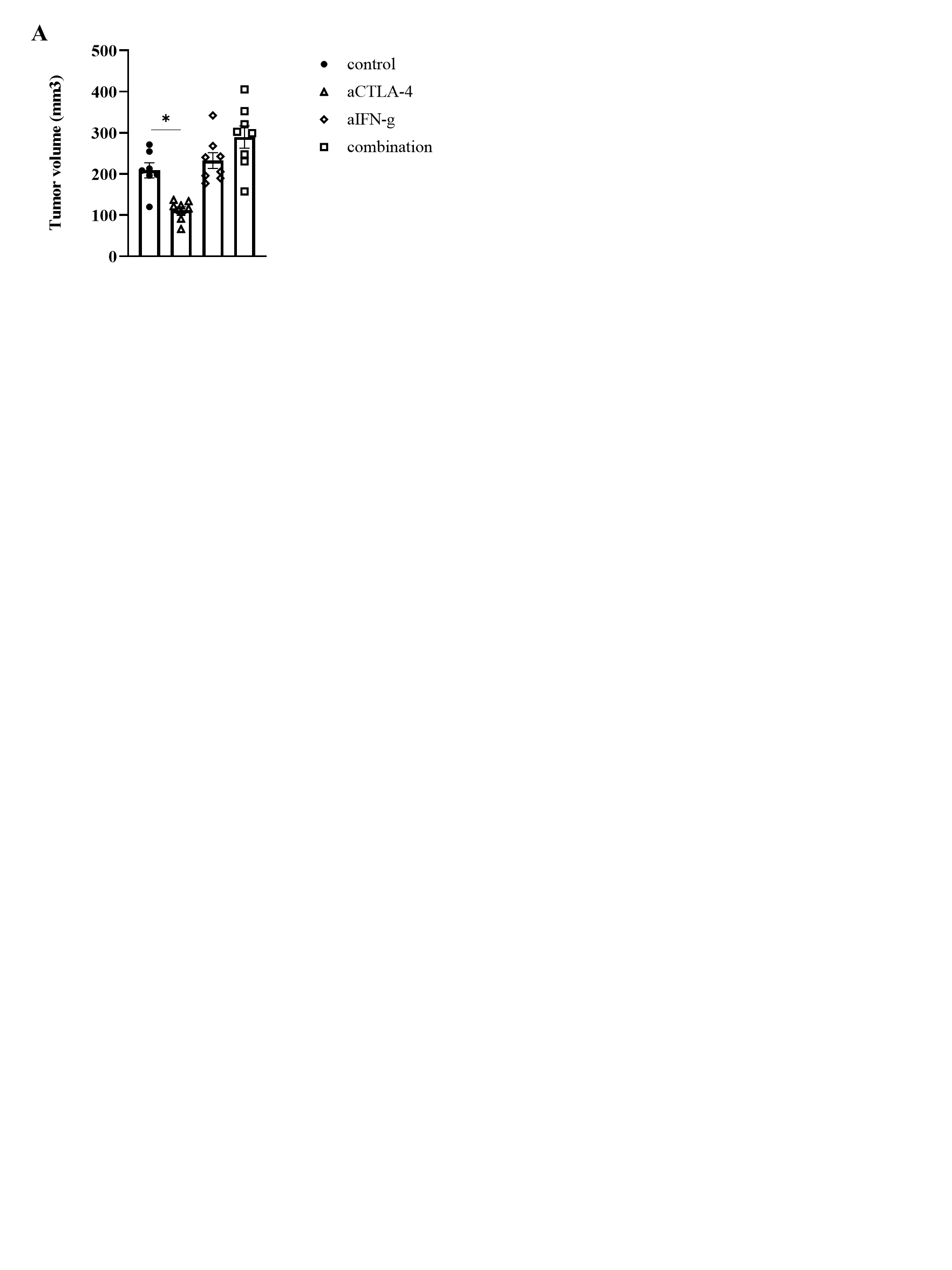

Supplement: S2 Fig — (A) Tumor growth of Hepa1-6 #12 tumors with or without anti-CTLA-4 Ab on days 5, 8, and 11 co-administered with an IFN-g depleting Ab on days 3, 7, and 10. Quantification of tumor volume at day 11. Data are shown as the mean ±SEM (n = 8). * P < 0.05 by one-way ANOVA followed by Dunnett’s test. (TIF) [file pone.0305984.s002.tif]

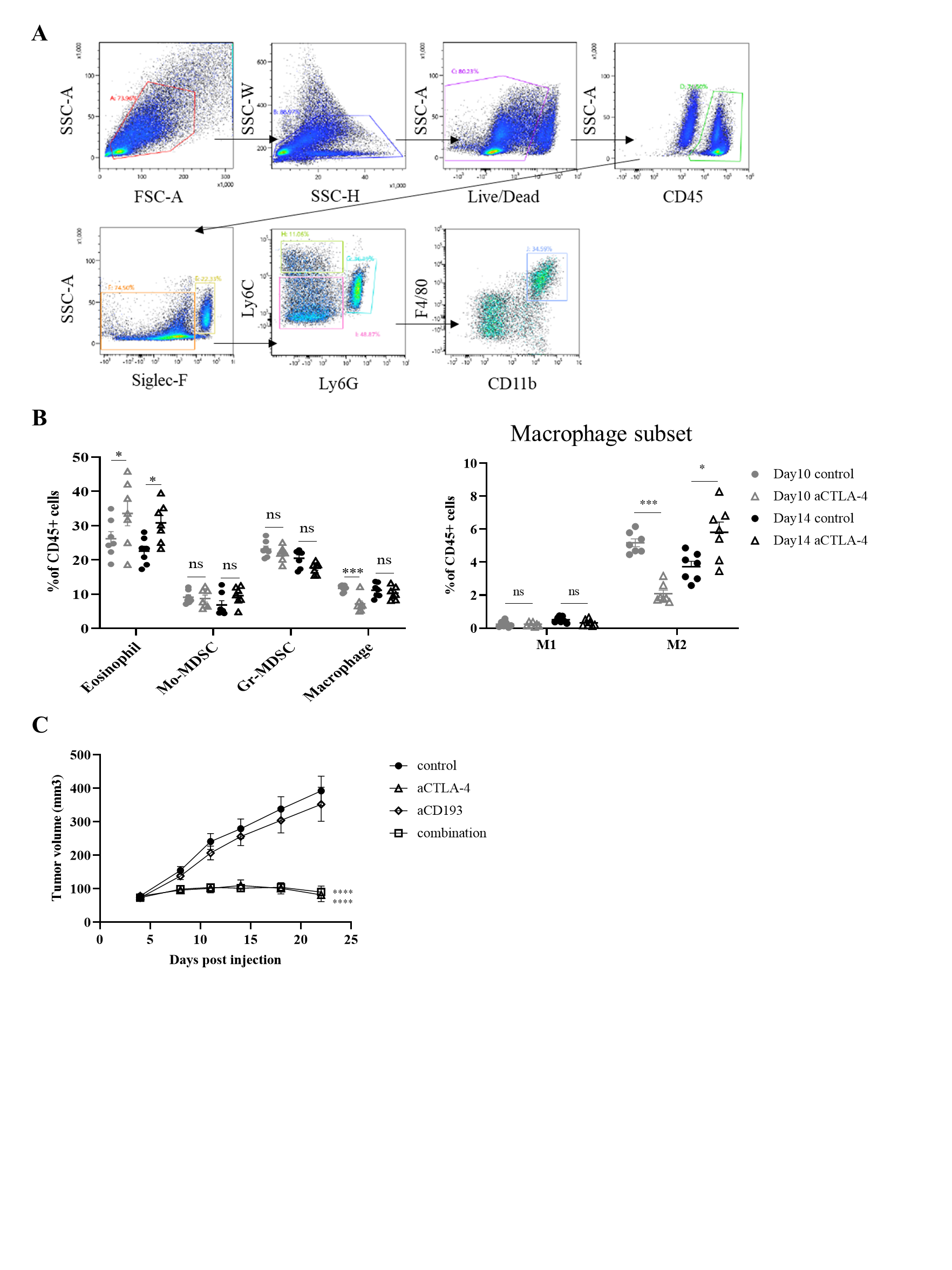

Supplement: S3 Fig — Tumors were treated with anti-CTLA-4 Ab or isotype control on days 5, 7, and 10. (A) Gating strategies to detect myeloid cells. (B) Tumor infiltrated myeloid cells were analyzed on days 10 and 14 by FCM. NOS2-producing macrophages are referred to as "M1" and arginase 1-producing macrophages are referred to as "M2". Percentage of each cell is shown as the mean ± SEM (n = 7). * P < 0.05, *** P < 0.001 by Mann-Whitney test. (C) Tumor growth of Hepa1-6 #12 tumors with or without anti-CTLA-4 Ab treatment on days 5, 8, 11, 14, and 18 co-administered with an eosinophil depleting Ab on days 4, 11, and 18. The average tumor volume is shown as the mean ± SEM (n = 7). **** P < 0.0001 by one-way ANOVA followed by Dunnett’s test at the endpoint. (TIF) [file pone.0305984.s003.tif]

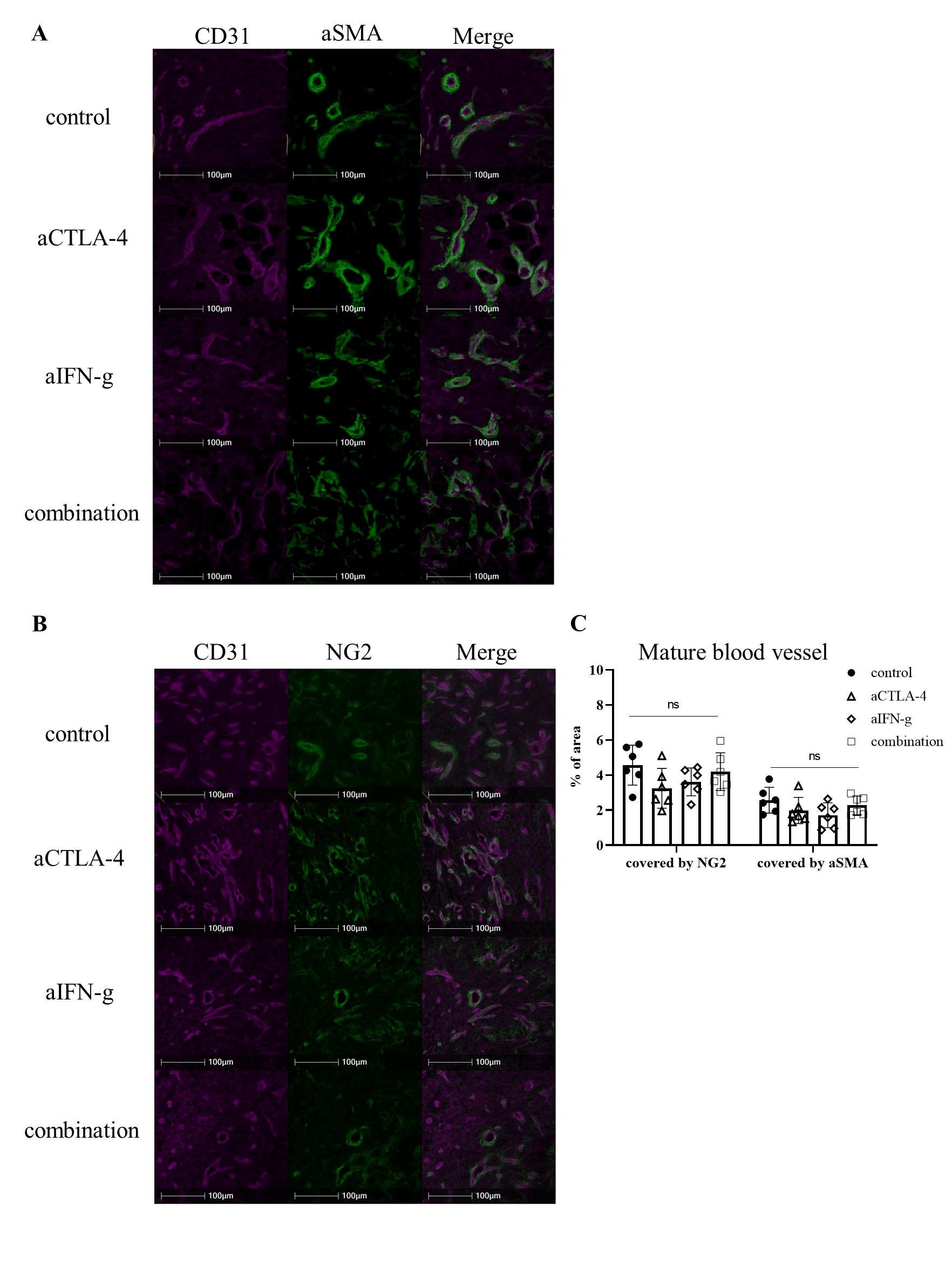

Supplement: S4 Fig — Tumors were treated with or without anti-CTLA-4 Ab treatment on days 5 and 8 co-administered with an IFN-g depleting Ab on days 4 and 7. Murine tumor sections were prepared on day 11 and analyzed using immunofluorescence staining for CD31, alpha-smooth muscle actin (α-SMA), and neuron-glial antigen 2 (NG2). Images were analyzed using HALO software. (A, B) Representative images are shown. (C) Quantification of mature blood vessel. Data are shown as the mean ± SEM (n = 6). * P < 0.05 by one-way ANOVA followed by Tukey’s test. (TIF) [file pone.0305984.s004.tif]

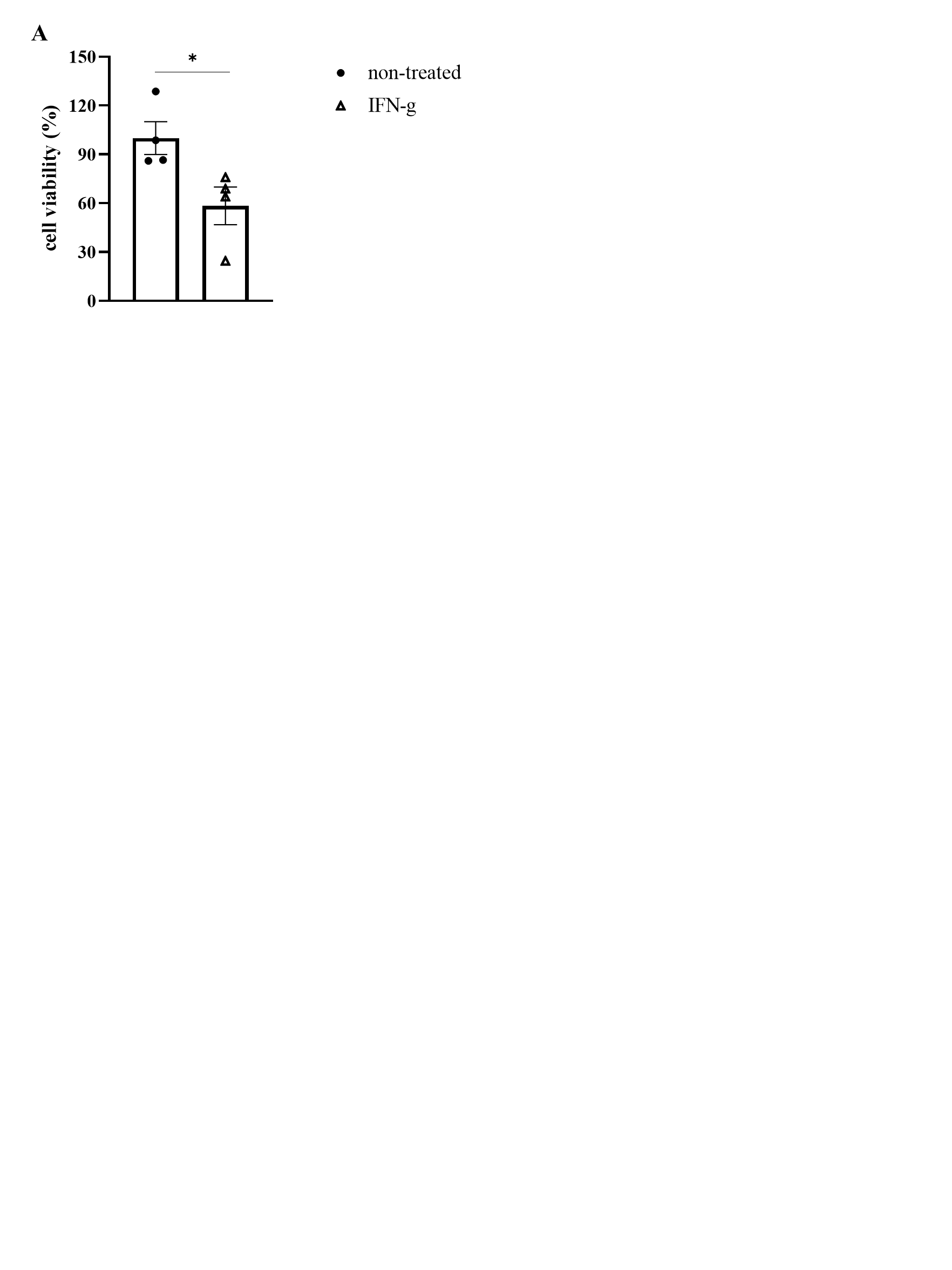

Supplement: S5 Fig — (A) Analysis of cell viability was performed by WST-8 assays after IFN-g addition. Data are shown as the mean ± SEM (n = 4). * P < 0.05 by Welch’s t-test. (TIF) [file pone.0305984.s005.tif]
